# Supplementary material for: Experimental study on the effect of Coumarone resin on the performance of SBS-modified asphalt
Source: PLoS One. 2024 Apr 18;19(4):e0301528. doi: 10.1371/journal.pone.0301528 (PMC11025964; doi:10.1371/journal.pone.0301528)
Supplement: Attachment — Submitted filename: response to Reviewers.docx [file pone.0301528.s002.docx]

**Response letter**

Dear Editors and Reviewers:

Sincerely thank you for your letter and reviewers’ comments concerning our manuscript entitled “Experimental study on the effect of Coumarone resin on the performance of SBS-modified asphalt” (PONE-D-23-42395). These valuable comments are very helpful for us to revise and improve the quality of the paper. We have studied comments carefully and made corrections which we hope meet with approval. Since the article underwent major changes, we have revised the Introduction, test program and Discussion section. Also, Some of the tables, figures and acknowledgment have also been updated (Table1 Line98, Table2 Line100, Table3 Line102, Fig.1 Line121, Fig.6 Line307, Fig.7 Line340, Fig.8 Line356). Revised portions are marked in blue in the manuscript. The corrections in the manuscript and the responses to reviewer’s comments are as following:

Reviewer 1

Question 1-Throughout the manuscript, the author shall use the same font for mentioning Degree Celsius.

Response 1

Thank you for your suggestion. We have changed the Degree Celsius in the manuscript to the same font. (Line112, Line138, Line469, Line470)

Question 2-L55: The authors used the abbreviation "C9", it is suggested to provide the full form for first time.

Response 2

Thank you very much for your advice.We have added the full form of C9 at Line55.

Question 3-In Table 1, 2, and 3, it is recommended to provide all units in brackets '()', instead of using '/'

Response 3

Thank you for your suggestion. We have modified all units in Table 1, 2 and 3 and put them in brackets '()'. (Line98, Line100, Line102)

Question 4-It is recommended to cite all the ASTM, AASHTO, and JTE specifications in proper reference style.

Response 4

Thank you very much for your advice.We refer to ASTM and AASHTO specifications for basic performance tests, DSR tests and segregation tests, respectively. (Line125, Line130, Line181)

Question 5- Section 2.8: Please provide chemical terms in proper form, i.e. In CCl4, 4 should be subscripted.

Response 5

Thank you very much for the correction, which we have corrected and marked in blue in the manuscript. (Line174, Line176)

Reviewer 2

Question 1-Please check the manuscript again.

Response 1

Thank you for your suggestion. We have again scrutinised the entire manuscript and found that the subheadings in Chapter 2 are inappropriately descriptive and that there are some narrative problems in the discussion section. We then revised it and marked it in blue in the manuscript and look forward to reviewing it again.

Question 2- Add an experimental plan for the manuscript.

Response 2

Thank you for your advice. We have made a flowchart of the experimental plan and put it in section 2.3. (Fig.1 Line121)

Question 3- Please add statistical comparison if possible, and error bars for all the bar charts in the manuscript.

Response 3

Thank you for your suggestion. We have reintroduced the bar charts with error bars into the manuscript and look forward to your review again. (Fig.6 Line307, Fig.7 Line340, Fig.8 Line356)

Question 4-The reasoning for the results is missing throughout the manuscript please add them.

Response 4

Thank you for your advice. We have now added more fully to the inferences of the results through further analysis of the experimental data and further study of the references, and the newly added exposition is marked blue in the manuscript and we hope to have your review. (Line253, Line296, Line381, Line392, Line451)
