# Supplementary material for: Experimental study on the effect of Coumarone resin on the performance of SBS-modified asphalt
Source: PLoS One. 2024 Apr 18;19(4):e0301528. doi: 10.1371/journal.pone.0301528 (PMC11025964; doi:10.1371/journal.pone.0301528)
Supplement: Attachment — Submitted filename: response to reviewers.docx [file pone.0301528.s003.docx]

**Response letter**

Dear Editors and Reviewers:

Sincerely thank you for your letter and reviewers’ comments concerning our manuscript entitled “Experimental study on the effect of Coumarone resin on the performance of SBS-modified asphalt” (PONE-D-23-42395). These valuable comments are very helpful for us to revise and improve the quality of the paper. We have studied comments carefully and made corrections which we hope meet with approval. Since the article underwent major changes, we have revised the Introduction, test program, Discussion and conclusion section. Revised portions are marked in blue in the manuscript. The corrections in the manuscript and the responses to reviewer’s comments are as following:

Reviewer 2

Question: Although all the comments are addressed carefully, kindly read the manuscript once again and fix the formatting discrepancies in the article. E.g.: Please capitalize the word flowchart in figure 1. The heading numbers are not formatted according to the journal requirements, Section 3.1.

Response

Thank you very much for your review and guidance, we have been in accordance with your requirements and revised the format of the manuscript, look forward to your review again, thank you! （LN134, LN207, LN506)

Reviewer 3

Question 1: The introduction section should be revised: More literature on coumarone resin-modified asphalt is required.

Response 1

Thank you very much for the strong support to our work. Through another literature search, we have placed additional articles on Coumarone resin-modified bitumen into the references. And the introductory section has been expanded and modified, and the modifications have been highlighted in blue font. Thank you again for your valuable suggestions. (LN73-80)

Question 2: LN 44 requires reference“However, the compatibility between SBS and asphalt is limited”. In addition, what did the authors mean by “compatibility”. Please further elaborate.

Response 2

Thank you for your advice. We have added a new reference to elucidate the compatibility of SBS polymers with asphalt. The compatibility of SBS with asphalt refers to the ability of the two to accommodate each other and form a macroscopically homogeneous modified asphalt. Detailed descriptions have been added to the manuscript and are highlighted in blue. Thank you for your suggestions to make the manuscript better. (LN44-47)

Question 3: LN 74-77: The research gap is unclear. Some studies already assess the coumarone resin performance when used in conjunction with asphalt. What does this study do differently?

Response 3

Thank you very much for your question, and I'm sorry that part was not well described by me. The studies that have been done on Coumarone resin and asphalt have investigated the effect of Coumarone resin on the properties of base asphalt, whereas these experiments of ours examined the effect of Coumarone resin on the properties of SBS-modified asphalt. The composite modification of both Coumarone resin and SBS is used to obtain modified asphalt with better performance. Specific amendments have been highlighted in blue in the manuscript. (LN84-88)

Question 4: LN85-87: what is road performance? Did you mean cracking resistance of flexible pavements? Or the rutting performance of AC? Please reframe the sentence for further clarity.

Response 4

Thank you for your advice. According to your suggestion, we describe the road performance of asphalt binder in detail as high temperature stability and low temperature cracking resistance. (LN95-97)

Question 5: LN95: Why did the authors select 4% SBS ratio?

Response 5

Thank you very much for your question, we realize that we have not expressed ourselves clearly here in writing about SBS modifier dosage. According to references and engineering experience, a 4% dosage of SBS in base asphalt can be used to represent the modified asphalt used in normal pavements, so there is a generalization in choosing a 4% dosage of SBS. We've made changes here in the manuscript and highlighted them in blue. (LN105-107)

Question 6: LN 126-127: Please provide the standards in references as well.

Response 6

Thank you for your valuable suggestions, we have added some reference indexes of SBS modified asphalt in the places you pointed out and highlighted them with blue fonts. (LN141-143)

Question 7: LN 145: Could you please provide details of short-term aging?

Response 7

Thank you very much for your advice. The short-term ageing of asphalt for this test was simulated using the rotating film oven test. Referring to the existing research results, 35g of unaged asphalt was filled into cylindrical specific glass bottles, which were placed into the turntable inside the RTFOT equipment, and the turntable was rotated at a speed of 15±0.2r/min for 85min at 163±0.5℃. The description has been added to the manuscript, thanks again for the suggestion. (LN160-164)

Question 8: LN 156: Please provide references.

Response 8

Thank you for your suggestion, we have added references to the manuscript about the BBR test. (LN175)

31. Xia PY, Hu LQ, Yan J, et al. Evaluation index of low temperature performance of high viscosity modified asphalt. China Sciencepaper, 2020, 15(12). (LN611)

Question 9: LN 191-194: How did authors conclude that the decrease in penetration is evidence of improvement in compatibility?

Response 9

Thank you very much for your question, and please apologize for not clearly expressing the reason for the drop in penetration. Due to the good compatibility of the resin, SBS and asphalt, the resin molecules enter the PS section of SBS effectively, thus strengthening the bond between the PS section of SBS and the asphalt. This is the reason for the increase in consistency and decrease in penetration of the modified asphalt, which we have rewritten in detail in the manuscript and highlighted in blue. (LN210-213)

Question 10: LN 221, please provide some references. Similarly, LN240-245. Also, to LN264.

Response 10

Thank you very much for your advice. We have added references below to the appropriate places in the manuscript and highlighted them in blue, so please review them again.

33. Walubita L F, Mahmoud E, Fuentes L, et al. Correlating the asphalt-binder high-temperature properties (DSR) to HMA permanent deformation (RLPD) and field rutting: A laboratory-field study. Construction and Building Materials, 2020, 262: 120761. (LN616)

35. Airey G D. Rheological properties of styrene butadiene styrene polymer modified road bitumens. Fuel, 2003, 82(14): 1709-1719. (LN621)

36. Meng YJ, Fan LP, Chen Q, et al. Microscopic Characteristics and Properties of Asphalt Modified by Waste Battery Powder. China Highway Journal, 2021, 34(10): 34-44. (LN623)

Question 11: Please also include limitations and future work in the manuscript.

Response 11

Thank you for your suggestion, we have included some of the limitations of this study and the possibility of future research on Coumarone resin in the conclusion, and we would appreciate your further review. (LN507-514)
